# Blood Hemoglobin, *in-vivo* Alzheimer Pathologies, and Cognitive Impairment: A Cross-Sectional Study

**DOI:** 10.3389/fnagi.2021.625511

**Published:** 2021-02-24

**Authors:** Jee Wook Kim, Min Soo Byun, Dahyun Yi, Jun Ho Lee, So Yeon Jeon, Kang Ko, Haejung Joung, Gijung Jung, Jun-Young Lee, Chul-Ho Sohn, Yun-Sang Lee, Yu Kyeong Kim, Dong Young Lee

**Affiliations:** ^1^Department of Neuropsychiatry, Hallym University Dongtan Sacred Heart Hospital, Hwaseong, South Korea; ^2^Department of Psychiatry, Hallym University College of Medicine, Chuncheon, South Korea; ^3^Department of Neuropsychiatry, Seoul National University Bundang Hospital, Seongnam, South Korea; ^4^Institute of Human Behavioral Medicine, Medical Research Center Seoul National University, Seoul, South Korea; ^5^Department of Geriatric Psychiatry, National Center for Mental Health, Seoul, South Korea; ^6^Department of Psychiatry, Chungnam National University Hospital, Daejeon, South Korea; ^7^Department of Neuropsychiatry, Seoul National University Hospital, Seoul, South Korea; ^8^Department of Neuropsychiatry, Seoul Metropolitan Government - Seoul National University Boramae Medical Center, Seoul, South Korea; ^9^Department of Radiology, Seoul National University Hospital, Seoul, South Korea; ^10^Department of Nuclear Medicine, Seoul National University College of Medicine, Seoul, South Korea; ^11^Department of Nuclear Medicine, Seoul Metropolitan Government - Seoul National University Boramae Medical Center, Seoul, South Korea; ^12^Department of Psychiatry, Seoul National University College of Medicine, Seoul, South Korea

**Keywords:** hemoglobin, anemia, Alzheimer's disease, cerebral hypometabolism, cognitive impairment

## Abstract

**Background:** Despite known associations between low blood hemoglobin level and Alzheimer's disease (AD) or cognitive impairment, the underlying neuropathological links are poorly understood. We aimed to examine the relationships of blood hemoglobin levels with *in vivo* AD pathologies (i.e., cerebral beta-amyloid [Aβ] deposition, tau deposition, and AD-signature degeneration) and white matter hyperintensities (WMHs), which are a measure of cerebrovascular injury. We also investigated the association between hemoglobin level and cognitive performance, and then assessed whether such an association is mediated by brain pathologies.

**Methods:** A total of 428 non-demented older adults underwent comprehensive clinical assessments, hemoglobin level measurement, and multimodal brain imaging, including Pittsburgh compound B-positron emission tomography (PET), AV-1451 PET, fluorodeoxyglucose (FDG)-PET, and magnetic resonance imaging. Episodic memory score and global cognition scores were also measured.

**Results:** A lower hemoglobin level was significantly associated with reduced AD-signature cerebral glucose metabolism (AD-CM), but not Aβ deposition, tau deposition, or WMH volume. A lower hemoglobin level was also significantly associated with poorer episodic memory and global cognition scores, but such associations disappeared when AD-CM was controlled as a covariate, indicating that AD-CM has a moderating effect.

**Conclusion:** The present findings suggest that low blood hemoglobin in older adults is associated with cognitive decline via reduced brain metabolism, which seems to be independent of those aspects of AD-specific protein pathologies and cerebrovascular injury that are reflected in PET and MRI measures.

## Introduction

Hemoglobin, a protein molecule present in red blood cells, contributes to the oxygen-carrying capacity of blood and related energy metabolism (Feig et al., 1971, 1972; Zauner et al., 2002; Schechter, 2008). Anemia, characterized by a decrease in the level of blood hemoglobin, is one of the most common blood disorders and has been reported to increase the risk of acute stroke (Tanne et al., 2010) and coronary heart disease (Astor et al., 2006; Mahmoodi et al., 2007). Many human studies have also found associations of low blood hemoglobin or anemia with increased risk of Alzheimer's disease (AD) (Shah et al., 2011; Faux et al., 2014; Wolters et al., 2019) or overall dementia (Atti et al., 2006; Hong et al., 2013; Wolters et al., 2019) and poorer cognitive performance (Shah et al., 2011).

Despite such associations of a lower blood hemoglobin level with AD and related cognitive impairment, the pathological mechanisms that underlie the associations are still poorly understood. A couple of preclinical and postmortem brain studies indicated that hemoglobin binds beta-amyloid protein (Aβ) and co-localizes with amyloid plaques (Oyama et al., 2000; Wu et al., 2004). Hemoglobin was also reported to alter the Aβ aggregation state (Chuang et al., 2012) and suppress Aβ-mediated inflammatory reactions (Sankar et al., 2018). A recent magnetic resonance imaging (MRI) study in a non-demented population showed an association of low blood hemoglobin level with altered white matter integrity and cerebral perfusion (Wolters et al., 2019). However, as of yet, no studies have investigated the relationship between blood hemoglobin and AD-specific pathologies in a living human brain.

Therefore, the present study was performed to investigate the associations between low blood hemoglobin and *in vivo* AD pathologies in non-demented older adults. We first examined the relationships of blood hemoglobin level with three AD pathologies (i.e., cerebral Aβ deposition, tau deposition, and AD-signature neurodegeneration) and white matter hyperintensities (WMHs), which are a measure of cerebrovascular injury. We then tried to examine whether the association of blood hemoglobin with cognitive impairment is affected by brain pathology which shows a significant relationship with lower hemoglobin level.

## Materials and Methods

### Participants

This study was part of the Korean Brain Aging Study for Early Diagnosis and Prediction of Alzheimer's Disease (KBASE), which is an ongoing prospective cohort study (Byun et al., 2017). As of February 2017, a total of 428 non-demented [289 cognitive normal (CN) and 139 mild cognitive impairment (MCI)] older adults between 55 and 90 years of age were enrolled in the study. The CN group consisted of participants with a Clinical Dementia Rating (CDR) (Morris, 1993) score of 0 and no diagnosis of MCI or dementia. All individuals with MCI met the current consensus criteria for amnestic MCI, which are as follows: (1) memory complaints confirmed by an informant; (2) objective memory impairments, (3) preserved global cognitive function; (4) independence in functional activities; and (5) no dementia. With regard to criterion 2, the age-, education-, and gender-adjusted z-scores for at least one of four episodic memory tests were < −1.0. The four memory tests were the Word List Memory, Word List Recall, Word List Recognition, and Constructional Recall tests, which are included in the Korean version of the Consortium to Establish a Registry for Alzheimer's Disease (CERAD-K) neuropsychological battery (Lee et al., 2004). All MCI individuals had a CDR score of 0.5. The exclusion criteria were as follows: (1) presence of a major psychiatric illness; (2) significant neurological (e.g., cerebrovascular disease) or medical conditions that could affect mental function; (3) contraindications for MRI (e.g., pacemaker or claustrophobia); (4) illiteracy; (5) the presence of significant visual/hearing difficulties and/or severe communication or behavioral problems that would make clinical examinations or brain scans difficult; (6) taking an investigational drug; and (7) pregnant or breastfeeding. The presence of any of the exclusion criteria was determined by research clinicians that referred to laboratory examination, MRI results, as well as clinical data collected by trained nurses during systematic interviews of participants and their reliable informants during the screening period. More detailed information on the recruitment of the KBASE cohort is presented in a previous report from our research group (Byun et al., 2017).

### Clinical Assessments

All participants underwent comprehensive clinical and neuropsychological assessments administered by trained psychiatrists and neuropsychologists based on the KBASE assessment protocol (Byun et al., 2017), which incorporates the Korean version of the CERAD neuropsychological battery (Morris et al., 1989; Lee et al., 2002, 2004). The episodic memory score (EMS) was calculated by summing the scores of the four episodic memory tests (i.e., Word List Memory, Word List Recall, Word List Recognition, and Constructional Recall) included in the CERAD neuropsychological battery. A CERAD total score (TS) was generated by summing the scores of seven tests in the CERAD neuropsychological battery including the Verbal Fluency, modified Boston Naming Test, Word List Memory, Constructional Praxis, Word List Recall, Word List Recognition, and Constructional recall tests (Seo et al., 2010). EMS and TS were selected as measures of episodic memory function and global cognitive function, respectively. Importantly, episodic memory decline is the earliest cognitive change observed in AD (Howieson et al., 1997; Grober et al., 2000). The comorbidity rates of vascular risk factors (e.g., hypertension, diabetes mellitus, dyslipidemia, coronary heart disease, transient ischemic attack, and stroke) were assessed based on data collected by trained nurses during systematic interviews of participants and their informants. A vascular risk score (VRS) was calculated based on the number of vascular risk factors present and reported as a percentage (Decarli et al., 2004). Body mass index (BMI) was calculated by dividing the weight in kilograms by the square of the height in meters. Annual income was evaluated and categorized into three groups [below the minimum cost of living (MCL), more than MCL but below twice the MCL, twice the MCL or more] (http://www.law.go.kr). Regarding occupational complexity, we considered only the longest-held occupation and then classified into four levels based on the skill levels described in International Standard Classification of Occupations (http://www.ilo.org/public/english/bureau/stat/isco/). The details of annual income and occupational complexity were described in Supplementary Material. Medication use within 4 weeks (no/yes), declined food intake over the past 3 months due to loss of appetite or swallowing difficulties (no/moderate/severe) (Vellas et al., 1999) smoking status (never/ex-smoker/smoker), and alcohol intake status (never/former/drinker) were also evaluated through nurse interviews and medical record review. To acquire accurate information, reliable informants were interviewed.

### Laboratory Tests of Blood Samples

After an overnight fast, blood samples were obtained via venipuncture in the morning (8–9 a.m.). Hemoglobin levels were measured using a flow cytometry method (ADVIA 2120i, Siemens, USA). The normal ranges for hemoglobin level are 12–15.5 g/dL in women and 13–17.5 g/dL in men (Shah et al., 2011). Serum creatinine levels were measured using a colorimetry method (ADVIA 1800 Auto Analyzer, Siemens, USA). Serum folate and vitamin B_12_ were measured using a radioimmunoassay method (Gamma-counter) with a vitamin B_12_ [^57^Co] / folate [^125^I] radioassay kit. Additionally, genomic DNA was extracted from whole blood and apolipoprotein E (*APOE*) genotyping was performed as previously described (Wenham et al., 1991). APOE ε4 (*APOE4*) positivity was defined as the presence of at least one ε4 allele.

### Measurement of Cerebral Aβ Deposition

All participants underwent simultaneous three-dimensional (3D) [^11^C] Pittsburgh compound B (PiB)-positron emission tomography (PET) and 3D T1-weighted MRI scanning using a 3.0T Biograph mMR (PET-MR) scanner (Siemens; Washington DC, WC, USA), in accordance with the manufacturer's guidelines. The details of the PiB-PET imaging acquisition and preprocessing were described previously (Park et al., 2019). An automatic anatomical labeling algorithm and a region-combining method(Reiman et al., 2009) were applied to determine regions of interest (ROIs) for characterization of PiB retention levels in the frontal, lateral parietal, posterior cingulate-precuneus, and lateral temporal regions. Standardized uptake value ratio (SUVR) values for each ROI were calculated by dividing the mean value for all voxels within each ROI by the mean cerebellar uptake value in the same image. A global cortical ROI consisting of the four ROIs was defined and a global Aβ retention value was generated by dividing the mean value for all voxels of the global cortical ROI by the mean cerebellar uptake value in the same image (Reiman et al., 2009; Choe et al., 2014). Participants were classified as Aβ-positive (Aβ+) if the SUVR value was > 1.4 in at least one of the four ROIs or as Aβ-negative (Aβ-) if the SUVR value was ≤ 1.4 for all four ROIs (Reiman et al., 2009; Jack et al., 2014).

### Measurement of Cerebral Tau Deposition

A subset of subjects (*n* = 107) underwent [^18^F] AV-1451 PET scans using a Biograph True point 40 PET/CT scanner (Siemens; Washington DC, WC, USA), in accordance with the manufacturer's guidelines. While all the other neuroimaging scans were performed during the baseline visit, AV-1451 PET imaging was performed at an average of 2.6 (standard deviation 0.3) years after the baseline visit. The details of AV-1451 PET imaging acquisition and preprocessing were described previously (Park et al., 2019). To estimate cerebral tau deposition, we quantified the AV-1541 SUVR of an a priori ROI of “AD-signature regions” of tau accumulation, which was composed of a size-weighted average of partial volume-corrected uptake in entorhinal, amygdala, parahippocampal, fusiform, inferior temporal, and middle temporal ROIs, in accordance with the method used in a previous report (Jack et al., 2017). The AV-1451 SUVR of the abovementioned ROI was used as an outcome variable for cerebral tau deposition.

### Measurement of AD-Signature Neurodegeneration

All participants underwent [^18^F] fluorodeoxyglucose (FDG)-PET imaging using the above-described PET-MR machine; the details of FDG-PET image acquisition and preprocessing were described previously (Park et al., 2019). AD-signature FDG ROIs that are sensitive to the changes associated with AD, such as the angular gyri, posterior cingulate cortex, and inferior temporal gyri (Jack et al., 2014), were determined. AD-signature cerebral glucose metabolism (AD-CM) was defined as the voxel-weighted mean SUVR extracted from the AD-signature FDG ROIs.

### Measurement of WMH

All participants underwent MRI scans including T1 weighted images and fluid-attenuated inversion recovery (FLAIR) images using the abovementioned 3.0T PET-MR machine. WMH volume was measured though a validated automatic procedure previously reported (Tsai et al., 2014). Briefly, the procedure consists of 11 steps: spatial co-registration of T1 and FLAIR images, fusion of T1 and FLAIR images, segmentation of T1 images, acquisition of transformation parameters, deformation and acquisition of the white matter mask, acquisition of FLAIR within the white matter mask, intensity normalization of the masked FLAIR, nomination of candidate WMH with a designated threshold, creation of a junction map, and elimination of the junction. There were two modifications in the current processing procedure relative to that used in the original study: (a) an optimal threshold of 70 was applied, as it was more suitable for our data than the threshold of 65 that was used in the original study; and, (b) given that individuals with acute cerebral infarcts were not enrolled in our sample, we did not use diffusion-weighted imaging in the current automated procedure. Using the final WMH candidate image, WMH volume was extracted in the native space in each subject.

### Statistical Analysis

To examine the relationships between hemoglobin level and neuroimaging biomarkers, multiple logistic, and linear regression analyses were performed as appropriate. Hemoglobin level, an independent variable for each analysis, was first entered as a continuous variable, and then as a stratified categorical variable. Subjects were divided into three strata [ <12 g/dL in female and <13 g/dL in male (anemia), ≤14 g/dL (low-normal level), and 14< g/dL (high-normal level)]. Within the normal range of hemoglobin level, the median value (i.e., 14 g/dL) was used as a cutoff to divide the low-normal and high-normal levels. To analyze the associations between hemoglobin and neuroimaging biomarkers, three models were tested for stepwise control of potential confounders. The first model did not include any covariates, the second model included age and sex as covariates, and the third model included all potential covariates (i.e., age, sex, education, *APOE4* positivity, VRS, clinical diagnosis, BMI, annual income status, occupational complexity, smoking, alcohol intake, vitamin B_12_, folate, platelet level, serum creatinine, medication use within 4 weeks, and declined food intake over past 3 months) that might confound the relationship between hemoglobin level and brain changes (Ballard, 1997; Vellas et al., 1999; Borroni et al., 2002; Chan and Mike, 2014; Shi et al., 2018). In multiple linear regression analyses, the normality was checked using the Kolmogorov-Smirnov test for dependent variable(s). While AD-CM was found to be normally distributed (statistic = 0.032, *df* = 420, p = 0.20), the other neuroimaging markers were not (statistic = 0.294, *df* = 420, p < 0.05 in global Aβ retention; statistic = 0.266, *df* = 106, *p* < 0.05 in AV-1451; and statistic = 0.139, *df* = 376, *p* < 0.05 in WMH). Therefore, the markers except AD-CM were used after natural log-transformation to achieve normal distribution. For the sensitivity analyses, the same analyses were performed for the subjects without cognitive impairment (i.e., CN subjects) or those without decreased food intake over the past 3 months due to loss of appetite or swallowing difficulties that could affect the hemoglobin level. For neuroimaging biomarkers that showed significant associations with hemoglobin level in the above analyses, further multiple linear regression analyses were performed that included hemoglobin×age (or sex or education or *APOE4* positivity or VRS or clinical diagnosis or BMI or annual income or occupational complexity) interaction term, as well as hemoglobin and age (or sex or education or *APOE4* positivity or VRS or clinical diagnosis or BMI or annual income or occupational complexity) as independent variables. In these analyses, the neuroimaging biomarker was used as a dependent variable, and the analyses were controlled for all potential covariates. To investigate the association between hemoglobin level and cognitive performance, a multiple linear regression model with hemoglobin as an independent variable and each of EMS and TS as a dependent variable was tested. Then, the same regression model was tested again while controlling for the neuroimaging biomarker that showed significant association with hemoglobin level as an additional covariate, to examine whether the relationship between blood hemoglobin and cognitive impairment is affected by the brain pathological marker. All statistical analyses were performed using IBM SPSS Statistics software (version 24, IBM Corp., Armonk, NY, USA).

## Results

### Participants

Demographic and clinical characteristics of the participants are presented in Table 1. Among the total of 428 participants, 183 had high or high-normal hemoglobin levels, 203 had low-normal hemoglobin levels, and 42 had low hemoglobin levels (anemia).

**Table 1:** Participant characteristics.

**Characteristic**	**Overall, *N* = 428**
Age, y	70.61 (8.01)
Female, *n* (%)	240 (56.07)
Education, y	11.18 (4.78)
APOE4 positivity, *n* (%)	99 (23.13)
Clinical diagnosis, CN, %	289 (67.52)
MMSE	25.45 (3.47)
**Hemoglobin level**	
Overall hemoglobin, g/dL	13.87 (1.30)
**Categorized hemoglobin**	
High, *n* (%)	2 (0.05)
High-normal, *n* (%)	181 (42.89)
Low-normal, *n* (%)	203 (47.43)
Anemia, *n* (%)	42 (9.81)
Red cell distribution width	12.89 (0.83)
Hematocrit	42.36 (3.87)
Iron (*n* = 376)	118.65 (39.14)
Transferrin (*n* = 376)	271.77 (40.65)
Ferritin (*n* = 376)	121.84 (92.87)
Body mass index, kg/m^2^	24.37 (3.03)
Vascular risk score, %	17.76 (16.33)
**Annual income status**	
<MCL, *n* (%)	35 (8.18)
≥MCL, <2 × MCL, *n* (%)	190 (44.39)
≥2 × MCL, *n* (%)	203 (47.43)
**Occupational complexity (*****N*** **= 427)**	
None, *n* (%)	78 (18.27)
Skill level 1, *n* (%)	29 (6.79)
Skill level 2, *n* (%)	141 (33.02)
Skill level 3, *n* (%)	56 (13.11)
Skill level 4, *n* (%)	123 (28.81)
**Smoking status**, ***n*** **(%)**	
Never/Former/ Smoker	287 (67.21)/119 (27.87)/21 (4.92)
**Alcohol intake status**, ***n*** **(%) (*****n*** **= 427)**	
Never/former/ drinker	231 (54.10)/57 (13.35)/139 (32.55)
Vitamin B_12_	580.93 (336.05)
Folic acid	10.01 (5.56)
Platelet, 10^9^/L	239.28 (57.49)
Serum creatinine, mg/dL	1.01 (0.20)
**Medication use within 4 weeks (*****n*** **= 427)**	
No, *n* (%)/Yes, *n* (%)	85 (19.91)/ 342 (80.09)
Declined food intake over past 3 months (n = 426)	
No, *n* (%)/Moderate, *n* (%)/Severe, *n* (%)	354 (83.10)/54 (12.68)/18 (4.23)
CERAD neuropsychological test, *z*-score	
Episodic memory score	−0.19 (1.21)
Memory total score	−0.19 (1.10)
Cognitive test score	−0.10 (0.86)
**AD neuroimage biomarkers**	
Cerebral Aβ deposition (*n* = 420)	
Aβ positivity, *n* (%)	101 (24.05)
Aβ retention, SUVR	11.28 (0.35)
**Cerebral tau deposition (*****n*** **= 106)**	
AV-1451, SUVR	1.60 (0.79)
AD-CM, SUVR (*n* = 420)	1.40 (0.13)
WMH volume, cm^3^ (*n* = 376)	5.99 (5.41)

*MMSE, mini-mental state examination; APOE4, apolipoprotein ε4; CN, cognitively normal; MCL, minimum cost of living; Aβ, beta-amyloid; AD, Alzheimer's disease; AD-CM, Alzheimer's disease signature cerebral glucose metabolism; SUVR, standardized uptake value ratio*.

*Unless otherwise indicated, data are expressed as mean (standard deviation)*.

### Association Between Hemoglobin Level and Neuroimaging Biomarkers

**Figure 1:**
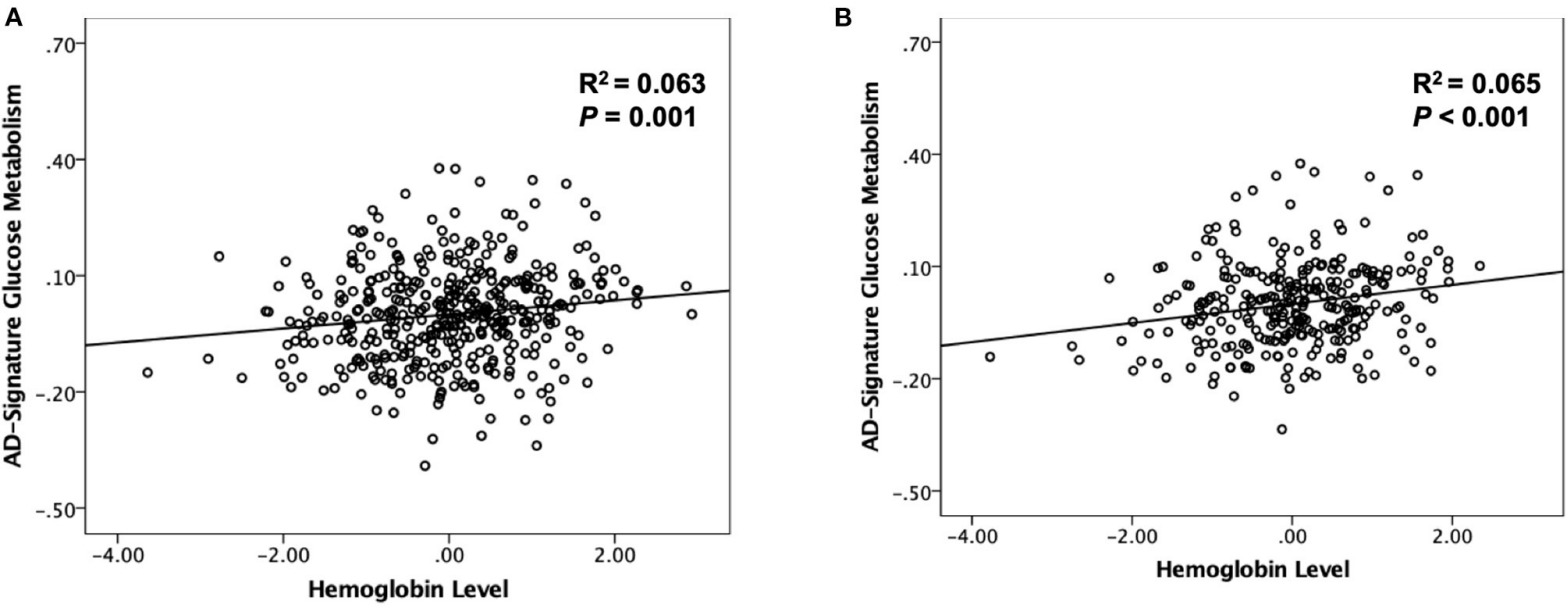
Partial regression plot showing the relationship between hemoglobin level and AD-signature cerebral glucose metabolism (AD-CM) in **(A)** non-demented and **(B)** cognitive normal older participants. Multiple linear regression analyses were performed after adjusting for age and sex.

**Figure 2:**
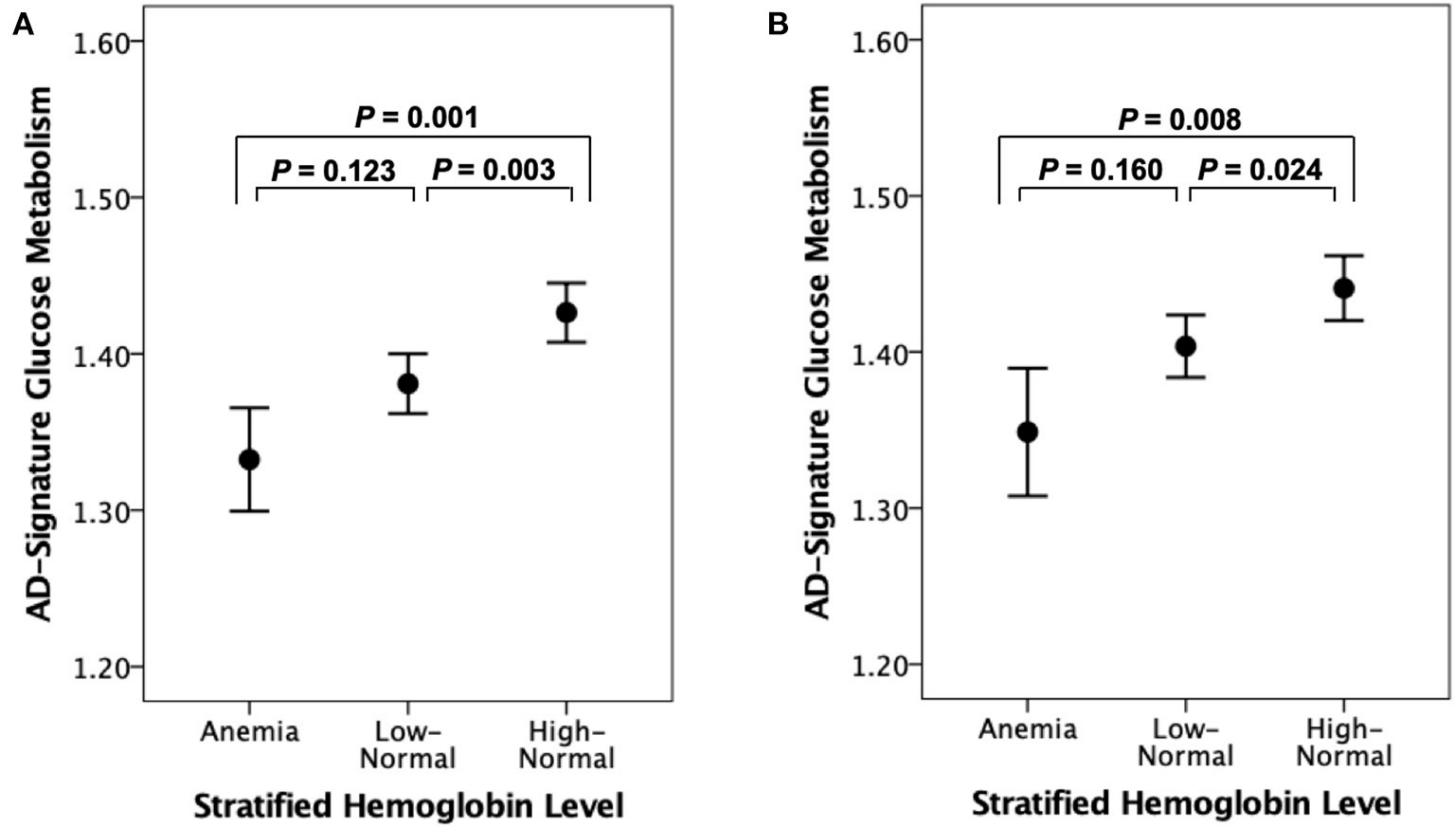
Error bar charts displaying AD-signature cerebral glucose metabolism and stratified hemoglobin level in **(A)** non-demented and **(B)** cognitive normal older participants. Error bars indicate standard error. Multiple linear regression analyses were performed after adjusting for age and sex.

As shown in Tables 2 and 3, neither Aβ biomarkers (i.e., Aβ positivity and Aβ deposition) nor tau deposition showed association with hemoglobin level (or strata) even after controlling for potential confounders. Hemoglobin level (or strata) was also not associated with WMH volume. In contrast, hemoglobin level (or strata) showed a significant positive association with AD-CM (Tables 2, 3 and Figures 1A, 2A). Both anemia and the low-normal stratum of hemoglobin showed significantly decreased AD-CM compared to the high-normal stratum. Sensitivity analyses that included only CN individuals produced very similar results for the relationships between hemoglobin and AD-CM (Supplementary Tables 1, 2; Figures 1B, 2B). Even when individuals without reduced food intake over the past 3 months were excluded, the results were not changed (Supplementary Tables 3, 4). Additional analyses to determine the moderation effects of age, sex, education, *APOE4* positivity, VRS, clinical diagnosis, BMI, annual income status, and occupational complexity on the association between hemoglobin and AD-CM did not reveal any significant results (Supplementary Table 5).

**Table 2:** Results of multiple logistic and linear regression analyses for assessing the relationship between hemoglobin level and Aβ, AV-1451, AD-CM, or WMH in non-demented older adults.

	**OR**	**95% CI**	***P***
**Aβ positivity**			
Model 1	0.816	0.684–0.974	0.024
Model 2	0.866	0.699–1.073	0.187
Model 3	0.894	0.682 to 1.171	0.416
	**B**	**95% CI**	***P***
**Aβ retention, SUVR**			
Model 1	−0.021	−0.038 to −0.004	0.014
Model 2	−0.012	−0.033 to 0.009	0.254
Model 3	−0.005	−0.025 to 0.014	0.590
**AV-1451, SUVR**			
Model 1	−0.020	−0.079 to 0.039	0.511
Model 2	−0.027	−0.097 to 0.042	0.439
Model 3	−0.038	−0.113 to 0.036	0.305
**AD-CM, SUVR**			
Model 1	0.019	0.010 to 0.029	< 0.001
Model 2	0.020	0.008 to 0.032	0.001
Model 3	0.018	0.006 to 0.030	0.004
**WMH, cm**^**3**^			
Model 1	−0.031	−0.105 to 0.042	0.404
Model 2	−0.015	−0.101 to 0.071	0.732
Model 3	−0.017	−0.110 to 0.077	0.722

*Aβ beta-amyloid; AD–CM Alzheimer's disease signature cerebral glucose metabolism; OR odds ratio; CI confidence interval*.

*The results of multivariate logistic or linear regression analyses are presented with OR or B coefficient values, 95% CI and P value*.

*Global Aβ retention, AV-1451, and WMH were used after natural log-transformation to achieve normal distribution*.

*Model 1 did not include any covariates, model 2 included age and sex as covariates, and model 3 included all potential covariates, including age, sex, education, apolipoprotein ε4, vascular risk score, clinical diagnosis, body mass index, annual income status, occupational complexity, smoking, alcohol intake, vitamin B_12_, folate, platelet level, serum creatinine, medication use, and declined food intake*.

**Table 3:** Results of multiple logistic and linear regression analyses for assessing the relationship between hemoglobin strata and Aβ, AV-1451, AD-CM, or WMH in non-demented older adults.

	**Stratified hemoglobin level**				
	**Anemia**		**Low-normal (≤14 g/dL)**		**High-normal (>14 g/dL)**
	**OR (95% CI)**	***P***	**OR (95% CI)**	***P***	
Aβ positivity					
Model 1	2.410 (1.153 to 5.037)	0.019	1.421 (0.868 to 2.326)	0.163	Reference
Model 2	1.649 (0.748 to 3.637)	0.215	1.216 (0.674 to 2.196)	0.515	Reference
Model 3	1.466 (0.541 to 3.977)	0.452	1.091 (0.554 to 2.151)	0.801	Reference
	**B (95% CI)**	***P***	**B (95% CI)**	***P***	
Aβ retention, SUVR					
Model 1	0.100 (0.022 to 0.179)	0.013	0.051 (0.005 to 0.098)	0.032	Reference
Model 2	0.062 (−0.019 to 0.143)	0.134	0.032 (−0.023 to 0.087)	0.249	Reference
Model 3	0.030 (−0.046 to 0.106)	0.442	0.020 (−0.028 to 0.068)	0.410	Reference
AV-1451, SUVR					
Model 1	0.095 (−0.174 to 0.364)	0.485	0.101 (−0.042 to 0.244)	0.165	Reference
Model 2	0.109 (−0.166 to 0.383)	0.433	0.165 (−0.019 to 0.348)	0.078	Reference
Model 3	0.112 (−0.179 to 0.403)	0.446	0.129 (−0.038 to 0.296)	0.129	Reference
AD-CM, SUVR					
Model 1	−0.092 (−0.136 to −0.048)	<0.001	−0.045 (−0.071 to −0.019)	0.001	Reference
Model 2	−0.081 (−0.127 to −0.036)	0.001	−0.047 (−0.078 to −0.016)	0.003	Reference
Model 3	−0.063 (−0.111 to −0.015)	0.010	−0.045 (−0.076 to −0.015)	0.003	Reference
WMH, cm^3^					
Model 1	0.141 (−0.189 to 0.471)	0.402	0.165 (−0.039 to 0.369)	0.112	Reference
Model 2	−0.006 (−0.339 to 0.327)	0.971	0.187 (−0.046 to 0.420)	0.116	Reference
Model 3	−0.028 (−0.392 to 0.337)	0.882	0.191 (−0.047 to 0.430)	0.116	Reference

*Aβ, beta-amyloid; AD-CM, Alzheimer's disease signature cerebral glucose metabolism; OR, odds ratio; CI, confidence interval*.

*Global Aβ retention, AV-1451, and WMH were used after natural log-transformation to achieve normal distribution*.

*Model 1 did not include any covariates, model 2 included age and sex as covariates, and model 3 included all potential covariates, including age, sex, education, apolipoprotein ε4, vascular risk score, clinical diagnosis, body mass index, annual income status, occupational complexity, smoking, alcohol intake, vitamin B_12_, folate, platelet level, serum creatinine, medication use, and declined food intake*.

### Association Between Hemoglobin and Cognition

**Figure 3:**
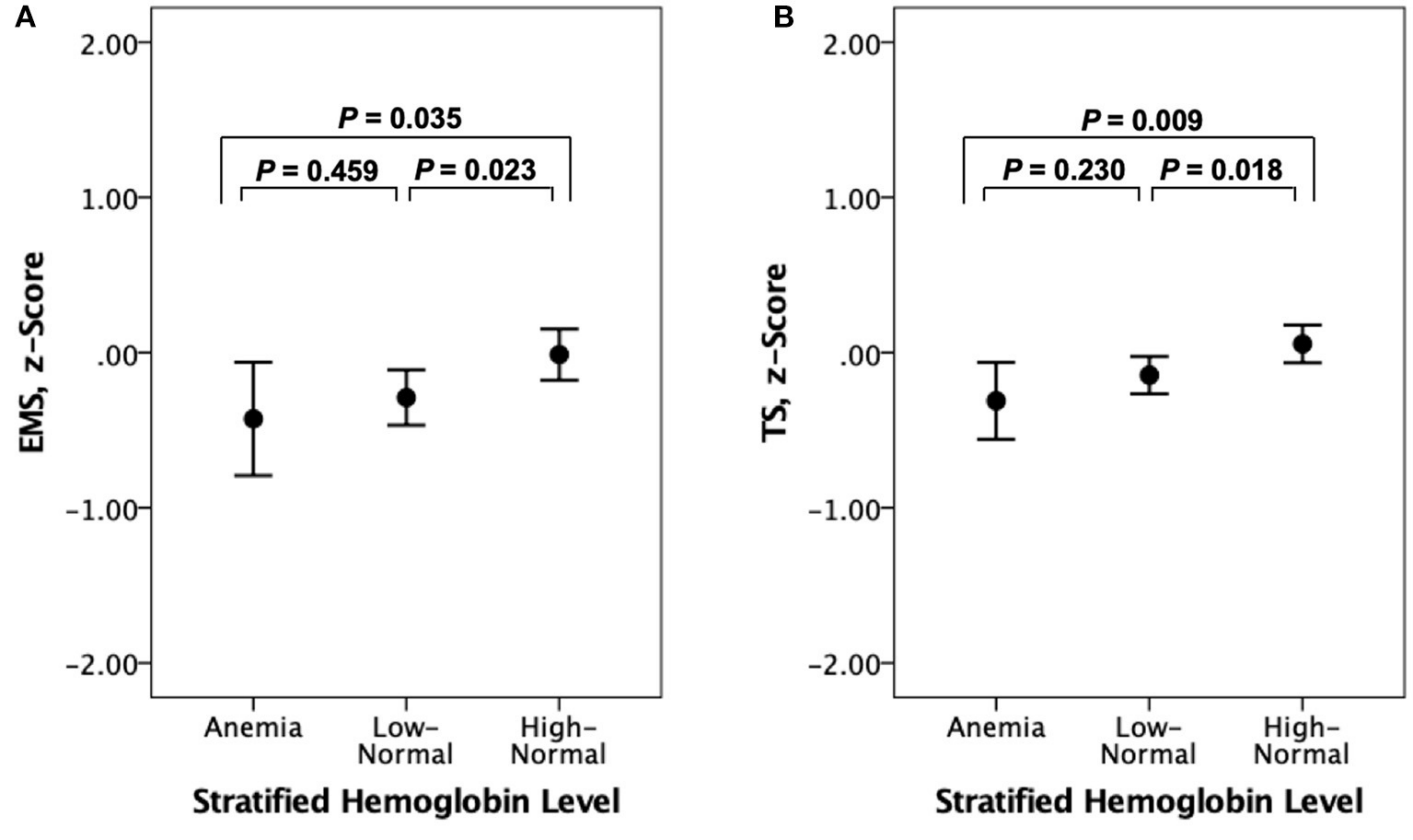
Error bar charts displaying **(A)** episodic memory score (EMS) and **(B)** CERAD total score (TS) according to stratified hemoglobin levels in non-demented older participants. Error bars indicate standard error.

Both EMS and TS were significantly different among the three hemoglobin strata (Table 4). The high-normal stratum had a significantly higher EMS and TS than the other two strata with lower hemoglobin levels (Figure 3). When AD-CM was controlled as an additional covariate, the relationship between hemoglobin strata and EMS or TS was no longer significant (Table 4).

**Table 4:** Results of multiple linear regression analyses for assessing the relationship between hemoglobin strata and cognitive performance in non-demented older adults.

	**Stratified hemoglobin level**				
	**Anemia**		**Low-normal (≤14 g/dL)**		**High-normal (**>**14 g/dL)**
	**B (95% CI)**	***P***	**B (95% CI)**	***P***	
EMS, *z*-score	−0.432 (−0.834 to −0.030)	0.035	−0.282 (−0.524 to −0.040)	0.023	Reference
EMS, *z*-score^†^	−0.266 (−0.670 to 0.138)	0.196	−0.202 (−0.443 to 0.040)	0.101	Reference
TS, *z*-score	−0.376 (−0.659 to −0.093)	0.009	−0.205 (−0.375 to −0.035)	0.018	Reference
TS, *z*-score^†^	−0.238 (−0.520 to 0.044)	0.098	−0.138 (−0.306 to 0.030)	0.108	Reference

*AD-CM, Alzheimer's disease signature cerebral glucose metabolism; CI, confidence interval; EMS, episodic memory score; TS, total score*.

† *To examine whether the association between hemoglobin and cognitive impairment is mediated by the brain pathological marker, we included AD-CM which showed a significant relationship with hemoglobin as an additional covariate in the multiple linear regression model with hemoglobin as an independent variable and each of EMS and TS as a dependent variable*.

## Discussion

In the present study, a lower blood hemoglobin was associated with reduced AD-CM, but not Aβ deposition, tau deposition or WMH volume, in non-demented older adults. There was also a significant association between lower hemoglobin and decreased cognitive performance. To the best of our knowledge, this is the first study to investigate the relationship between hemoglobin and AD-specific pathologies in the living human brain.

We found a strong association between anemia or a low-normal level of hemoglobin and decreased AD-CM. The present findings are in line with previous reports that found a strong relationship between a low level of hemoglobin and AD dementia (Shah et al., 2011; Wolters et al., 2019). Regarding the association between lower hemoglobin and deceased AD-CM, there are a couple of possible explanations. First, lower hemoglobin or anemia itself can decrease the delivery of oxygen to brain tissues (Kosenko et al., 2017), and cause oxidative damage to key enzymes involved in glycolysis, the tricarboxylic acid cycle and adenosine triphosphate (ATP) biosynthesis (Butterfield and Halliwell, 2019). This damage to energy-related processes negatively affects the metabolism of glucose, a key source of energy for the brain, and subsequently results in the characteristic reduction of cerebral glucose metabolism that is found in AD dementia (Aliev et al., 2003; Perry et al., 2003; Butterfield and Halliwell, 2019). Second, hemoglobin may also be lowered by decreased oral intake and poor nutrition in individuals with cognitive impairment. However, this possibility seems very low since the sensitivity analysis for CN subjects or those without declined food intake over the past 3 months revealed similar findings. Additionally, lower socio-economic status (SES) may just mediate the association between lower hemoglobin and deceased AD-CM because it could be related with not only low hemoglobin level via chronic poor nutritional intake and limited access to medical care, but also reduced brain metabolism and poor cognition (Farah, 2017). Given that the result did not change even after controlling annual income and occupational complexity as indicators for SES as covariates (in Model 3), however, this possibility appears very low.

Although several preclinical and postmortem studies showed a possible association between hemoglobin and Aβ pathology itself or its downstream pathological changes (Oyama et al., 2000; Wu et al., 2004), we could not find any association of hemoglobin with Aβ or tau pathologies. This indicates that AD-specific protein pathologies are not associated with lower hemoglobin and dementia or cognitive decline. Additionally, WMH volume, a measure of cerebrovascular injury, was not associated with hemoglobin in the present study. This finding is not in line with the result from a recent human study, which reported that a lower level of hemoglobin was associated with increased WMH volume in non-demented individuals (Wolters et al., 2019). This discrepancy may be explained by differences in the characteristics of study participants regarding the presence of severe cerebrovascular disease. While the aforementioned study included individuals with severe cerebrovascular lesions, the present study did not. The null finding in the current study may be related to the low variability or relatively low burden of WMH or cerebrovascular injury.

We also found a relationship between low hemoglobin and poorer episodic memory or overall cognition, consistent with previous similar observations (Deal et al., 2009; Shah et al., 2011; Qin et al., 2019). When AD-CM, which showed significant association with hemoglobin, was adjusted as an additional covariate, the positive relationship between hemoglobin level and cognitive function was no longer significant. These findings further support the possibility that low hemoglobin or anemia may contribute to the development of AD dementia and related cognitive decline via cerebral hypometabolism.

There were a few limitations in the present study. First, because this was a cross-sectional study, causal relationships cannot be easily inferred from the findings. Long term prospective studies are still needed to confirm the etiological contribution of low hemoglobin. Second, we did not assess the relationships between an abnormally high level of hemoglobin (<15.5 g/dL in female and <17.5 g/dL in male) and neuroimaging markers or cognition, although such high levels of hemoglobin, as well as anemia, have been reported to increase the risk of poor cognitive performance (Shah et al., 2011). This was because only two subjects had a high level of hemoglobin in the present study. Further studies that include individuals within the entire range of possible hemoglobin levels will be helpful to obtain a more comprehensive understanding of the association between hemoglobin and brain pathologies or related cognitive impairment. Third, tau PET was applied after an average of 2.6 years from the baseline visit, whereas other neuroimaging scans were performed at baseline. This temporal gap may have influenced the association between hemoglobin and tau. When we controlled for the temporal gap as an additional covariate, however, the results did not change. In addition, only a subset of participants (*n* = 107) underwent tau PET, while all participants underwent the other imaging modalities. This relatively reduced sample size for tau PET may have decreased the statistical power and contributed to the null result for the relationship between hemoglobin and tau deposition. A study with a larger sample size is still needed to confirm this finding. Lastly, history of vascular risk factors, included as one of the covariates in the Model 3, was assessed only based on the data collected through systematic interviews of participants and their informants. Detailed review of medical record or use of direct physiological or biochemical measurements may help to obtain more accurate information.

## Conclusion

The present findings suggest that low blood hemoglobin in older adults is associated with cognitive decline via reduced brain metabolism, which seems to be independent of those aspects of AD-specific protein pathologies and cerebrovascular injury that are reflected in PET and MRI measures.

## Data Availability Statement

The data of the current study can be available from the independent data sharing committee of the KBASE research group on reasonable request. Requests for data access can be submitted to the administrative coordinator of the KBASE group by e-mail (kbasecohort@gmail.com).

## Ethics Statement

This study was approved by the Institutional Review Boards of Seoul National University Hospital (IRB No: C-1401-027547) and SNU-SMG Boramae Center (IRB No: 26-2015-60), Seoul, South Korea, and was conducted in accordance with the recommendations of the current version of the Declaration of Helsinki. All subjects or their legal representatives gave written and informed consent.

## Author Contributions

JK and DL conceived and designed the study. MB, DY, JL, SJ, KK, HJ, GJ, J-YL, C-HS, Y-SL, YK, and DL were involved in acquisition, or analysis and interpretation of the data and helped to draft the manuscript. JK, MB, DY, JL, and DL were major contributors in writing the manuscript and critically revising the manuscript for intellectual content. DL served as principal investigator and supervised the study. All authors read and approved the final manuscript.

## Conflict of Interest

The authors declare that the research was conducted in the absence of any commercial or financial relationships that could be construed as a potential conflict of interest.
