# Supplementary material for: Blood Hemoglobin, in-vivo Alzheimer Pathologies, and Cognitive Impairment: A Cross-Sectional Study
Source: Front Aging Neurosci. 2021 Feb 24;13:625511. doi: 10.3389/fnagi.2021.625511 (PMC7943867; doi:10.3389/fnagi.2021.625511)
Supplement: Supplementary file 1 [file Table_1.docx]

**SUPPLEMENTARY INFORMATION**

**STROBE CHECKLIST**. Strengthening the Reporting of Observational Studies in Epidemiology (STROBE) Checklist.

**SUPPLEMENTARY METHODS.** Assessment of annual income and occupational complexity.

**SUPPLEMENTARY** **TABLE 1.** Results of multiple logistic and linear regression analyses for assessing the relationship between hemoglobin level and Aβ, AV-1451, AD-CM, WMH in cognitive normal older adults

**SUPPLEMENTARY** **TABLE 2.** Results of multiple logistic and linear regression analyses for assessing the relationship between hemoglobin strata and Aβ, AV-1451, AD-CM, or WMH in cognitive normal older adults.

**SUPPLEMENTARY** **TABLE 3.** Results of multiple logistic and linear regression analyses for assessing the relationship between hemoglobin level and Aβ, AV-1451, AD-CM, WMH in non-demented older adults without reduced food intake.

**SUPPLEMENTARY** **TABLE 4.** Results of multiple logistic and linear regression analyses for assessing the relationship between hemoglobin strata and Aβ, AV-1451, AD-CM, or WMH in non-demented older adults without reduced food intake.

**SUPPLEMENTARY** **TABLE 5.** Results of multiple linear regression analyses including the interaction term between hemoglobin and age (or sex or education or apolipoprotein ε4 positivity or vascular risk score or clinical diagnosis or body mass index or annual income or occupational complexity) status predicting AD-CM for non-demented older subjects.

This supplementary material has been provided by the authors to give readers additional information about their work.

**SUPPLEMENTARY METHODS.** Assessment of annual income and occupational complexity.

Annual income was evaluated and categorized into three groups [below the minimum cost of living (MCL), more than MCL but below twice the MCL, twice the MCL or more] (http://www.law.go.kr). The MCL was determined according to the administrative rule published by the Ministry of Health and Welfare, Republic of Korea in November 2012. The MCL was 572,168 Korean Won (KRW) [equivalent to 507.9 US dollar (USD)] per month for single-person household and added 286,840 KRW (equivalent to 254.6 USD) per month for each additional housemate. Regarding occupational complexity, we considered only the longest-held occupation and then classified into four levels based on the skill levels described in International Standard Classification of Occupations (<http://www.ilo.org/public/english/bureau/stat/isco/>). Occupations at skill level 1 typically require simple and routine physical or manual tasks. Occupations at skill level 2 involve the performance of tasks, such as operating machinery and electronic equipment, driving vehicles, maintenance and repair of electrical and mechanical equipment and manipulation, ordering and storage of information. Occupations at skill level 3 include the performance of complex technical and practical tasks that require complex problem solving, reasoning, and decision making in a specialized field. Occupations at skill level 4 involve the performance of tasks that require complex problem-solving, decision-making and creativity based on an extensive body of theoretical and factual knowledge in a specialized field. Information about occupation was obtained from self-report by the participants and confirmed by reliable informants.

| **SUPPLEMENTARY** **TABLE 1.** Results of multiple logistic and linear regression analyses for assessing the relationship between hemoglobin level and Aβ, AV-1451, AD-CM, WMH in cognitive normal older adults | | | |
| --- | --- | --- | --- |
|  | OR | 95% CI | *P* |
| Aβ positivity |  |  |  |
| Model 1 | 0.903 | -0.693 to 1.176 | 0.448 |
| Model 2 | 0.939 | 0.671 to 1.314 | 0.714 |
| Model 3 | 0.963 | 0.648 to 1.431 | 0.852 |
|  |  |  |  |
|  | B | 95% CI | *P* |
| Aβ retention, SUVR |  |  |  |
| Model 1 | -0.011 | -0.026 to 0.004 | 0.145 |
| Model 2 | -0.008 | -0.027 to 0.011 | 0.385 |
| Model 3 | -0.010 | -0.029 to 0.010 | 0.338 |
| AV-1451, SUVR |  |  |  |
| Model 1 | -0.012 | -0.049 to 0.024 | 0.502 |
| Model 2 | -0.018 | -0.062 to 0.027 | 0.428 |
| Model 3 | -0.003 | -0.062 to 0.055 | 0.914 |
| AD-CM, SUVR |  |  |  |
| Model 1 | 0.020 | 0.010 to 0.031 | <0.001 |
| Model 2 | 0.026 | 0.012 to 0.040 | <0.001 |
| Model 3 | 0.027 | 0.012 to 0.041 | <0.001 |
| WMH, cm^3^ |  |  |  |
| Model 1 | -0.042 | -0.133 to 0.050 | 0.372 |
| Model 2 | -0.044 | -0.158 to 0.071 | 0.454 |
| Model 3 | -0.045 | -0.167 to 0.078 | 0.471 |
| Abbreviations: *Aβ* beta-amyloid; *AD-CM* Alzheimer’s disease signature cerebral glucose metabolism; *OR* odds ratio; *CI* confidence interval.  The results of multivariate logistic or linear regression analyses are presented with OR or B coefficient values, 95% CI and *P* value.  Global Aβ retention, AV-1451, and WMH were used after natural log-transformation to achieve normal distribution.  Model 1 did not include any covariates, model 2 included age and sex as covariates, and model 3 included all potential covariates, including age, sex, education, apolipoprotein ε4 positivity, vascular risk score, body mass index, annual income status, occupational complexity, smoking, alcohol intake, vitamin B_12_, folate, platelet level, serum creatinine, medication use, and declined food intake. | | | |

| **SUPPLEMENTARY** **TABLE 2.** Results of multiple logistic and linear regression analyses for assessing the relationship between hemoglobin strata and Aβ, AV-1451, AD-CM, or WMH in cognitive normal older adults. | | | | | |
| --- | --- | --- | --- | --- | --- |
|  | Stratified hemoglobin level | | | | |
|  | Anemia | | Low-normal  ($\leq$14 g/dL) | | High-normal  ($>$14 g/dL) |
|  | OR (95% CI) | *P* | OR (95% CI) | *P* |  |
| Aβ positivity |  |  |  |  |  |
| Model 1 | 1.311 (0.345 to 4.983) | 0.691 | 1.122 (0.560 to 2.248) | 0.745 | Reference |
| Model 2 | 0.675 (0.159 to 2.877) | 0.596 | 0.922 (0.377 to 2.260) | 0.860 | Reference |
| Model 3 | 0.592 (0.113 to 3.097) | 0.535 | 0.755 (0.291 to 1.958) | 0.563 | Reference |
|  |  |  |  |  |  |
|  | B (95% CI) | *P* | B (95% CI) | *P* |  |
| Aβ retention, SUVR |  |  |  |  |  |
| Model 1 | 0.026 (-0.054 to 0.106) | 0.522 | 0.024 (-0.015 to 0.063) | 0.230 | Reference |
| Model 2 | -0.002 (-0.084 to 0.080) | 0.962 | 0.013 (-0.034 to 0.061) | 0.575 | Reference |
| Model 3 | 0.006 (-0.079 to 0.092) | 0.883 | 0.007 (-0.040 to 0.055) | 0.755 | Reference |
| AV-1451, SUVR |  |  |  |  |  |
| Model 1 | 0.114 (-0.049 to 0.277) | 0.167 | 0.049 (-0.034 to 0.132) | 0.246 | Reference |
| Model 2 | 0.109 (-0.056 to 0.273) | 0.191 | 0.068 (-0.037 to 0.173) | 0.200 | Reference |
| Model 3 | 0.038 (-0.181 to 0.258) | 0.726 | 0.047 (-0.077 to 0.171) | 0.451 | Reference |
| AD-CM, SUVR |  |  |  |  |  |
| Model 1 | -0.086 (-0.144 to -0.029) | 0.003 | -0.037 (-0.065 to -0.009) | 0.010 | Reference |
| Model 2 | -0.082 (-0.142 to -0.022) | 0.008 | -0.040 (-0.074 to -0.005) | 0.024 | Reference |
| Model 3 | -0.072 (-0.135 to -0.008) | 0.027 | -0.040 (-0.075 to -0.005) | 0.025 | Reference |
| WMH, cm^3^ |  |  |  |  |  |
| Model 1 | 0.133 (-0.343 to 0.608) | 0.583 | 0.221 (-0.022 to 0.464) | 0.075 | Reference |
| Model 2 | 0.023 (-0.460 to 0.505) | 0.927 | 0.262 (-0.025 to 0.549) | 0.074 | Reference |
| Model 3 | 0.018 (-0.506 to 0.541) | 0.947 | 0.260 (-0.037 to 0.557) | 0.085 | Reference |
| Abbreviations: *Aβ* beta-amyloid; *AD-CM* Alzheimer’s disease signature cerebral glucose metabolism; *OR* odds ratio; *CI* confidence interval.  Global Aβ retention, AV-1451, and WMH were used after natural log-transformation to achieve normal distribution.  Model 1 did not include any covariates, model 2 included age and sex as covariates, and model 3 included all potential covariates, including age, sex, education, apolipoprotein ε4 positivity, vascular risk score, body mass index, annual income status, occupational complexity, smoking, alcohol intake, vitamin B_12_, folate, platelet level, serum creatinine, medication use, and declined food intake. | | | | | |

| **SUPPLEMENTARY** **TABLE 3.** Results of multiple logistic and linear regression analyses for assessing the relationship between hemoglobin level and Aβ, AV-1451, AD-CM, WMH in non-demented older adults without reduced food intake. | | | |
| --- | --- | --- | --- |
|  | OR | 95% CI | *P* |
| Aβ positivity |  |  |  |
| Model 1 | 0.804 | 0.656 to 0.986 | 0.036 |
| Model 2 | 0.836 | 0.649 to 1.078 | 0.167 |
| Model 3 | 0.914 | 0.669 to 1.249 | 0.573 |
|  |  |  |  |
|  | B | 95% CI | *P* |
| Aβ retention, SUVR |  |  |  |
| Model 1 | -0.022 | -0.041 to -0.002 | 0.032 |
| Model 2 | -0.012 | -0.036 to 0.013 | 0.356 |
| Model 3 | -0.004 | -0.027 to 0.019 | 0.729 |
| AV-1451, SUVR |  |  |  |
| Model 1 | -0.027 | -0.093 to 0.040 | 0.422 |
| Model 2 | -0.029 | -0.112 to 0.055 | 0.498 |
| Model 3 | -0.057 | -0.137 to 0.024 | 0.166 |
| AD-CM, SUVR |  |  |  |
| Model 1 | 0.018 | 0.007 to 0.029 | 0.001 |
| Model 2 | 0.019 | 0.006 to 0.033 | 0.006 |
| Model 3 | 0.020 | 0.006 to 0.034 | 0.006 |
| WMH, cm^3^ |  |  |  |
| Model 1 | 0.004 | -0.082 to 0.089 | 0.928 |
| Model 2 | 0.001 | -0.102 to 0.104 | 0.981 |
| Model 3 | -0.015 | -0.126 to 0.096 | 0.791 |
| Abbreviations: *Aβ* beta-amyloid; *AD-CM* Alzheimer’s disease signature cerebral glucose metabolism; *OR* odds ratio; *CI* confidence interval.  The results of multivariate logistic or linear regression analyses are presented with OR or B coefficient values, 95% CI and *P* value.  Global Aβ retention, AV-1451, and WMH were used after natural log-transformation to achieve normal distribution.  Model 1 did not include any covariates, model 2 included age and sex as covariates, and model 3 included all potential covariates, including age, sex, education, apolipoprotein ε4 positivity, vascular risk score, body mass index, annual income status, occupational complexity, smoking, alcohol intake, vitamin B_12_, folate, platelet level, serum creatinine, and medication use. | | | |

| **SUPPLEMENTARY** **TABLE 4.** Results of multiple logistic and linear regression analyses for assessing the relationship between hemoglobin strata and Aβ, AV-1451, AD-CM, or WMH in non-demented older adults without reduced food intake. | | | | | |
| --- | --- | --- | --- | --- | --- |
|  | Stratified hemoglobin level | | | | |
|  | Anemia | | Low-normal  ($\leq$14 g/dL) | | High-normal  ($>$14 g/dL) |
|  | OR (95% CI) | *P* | OR (95% CI) | *P* |  |
| Aβ positivity |  |  |  |  |  |
| Model 1 | 2.287 (0.922 to 5.678) | 0.074 | 1.422 (0.837 to 2.414) | 0.193 | Reference |
| Model 2 | 1.679 (0.636 to 4.431) | 0.295 | 1.248 (0.659 to 2.362) | 0.496 | Reference |
| Model 3 | 1.306 (0.399 to 4.269) | 0.659 | 1.009 (0.489 to 2.080) | 0.982 | Reference |
|  |  |  |  |  |  |
|  | B (95% CI) | *P* | B (95% CI) | *P* |  |
| Aβ retention, SUVR |  |  |  |  |  |
| Model 1 | 0.084 (-0.015 to 0.184) | 0.095 | 0.059 (0.007 to 0.111) | 0.025 | Reference |
| Model 2 | 0.051 (-0.052 to 0.153) | 0.331 | 0.041 (-0.019 to 0.102) | 0.178 | Reference |
| Model 3 | 0.020 (-0.075 to 0.116) | 0.673 | 0.026 (-0.028 to 0.080) | 0.339 | Reference |
| AV-1451, SUVR |  |  |  |  |  |
| Model 1 | 0.062 (-0.341 to 0.465) | 0.761 | 0.096 (-0.048 to 0.240) | 0.190 | Reference |
| Model 2 | 0.070 (-0.345 to 0.485) | 0.738 | 0.129 (-0.062 to 0.319) | 0.182 | Reference |
| Model 3 | 0.148 (-0.225 to 0.522) | 0.431 | 0.145 (-0.029 to 0.320) | 0.101 | Reference |
| AD-CM, SUVR |  |  |  |  |  |
| Model 1 | -0.081 (-0.136 to -0.026) | 0.004 | -0.048 (-0.077 to -0.019) | 0.001 | Reference |
| Model 2 | -0.074 (-0.131 to -0.017) | 0.011 | -0.052 (-0.085 to -0.018) | 0.003 | Reference |
| Model 3 | -0.067 (-0.126 to -0.008) | 0.027 | -0.049 (-0.082 to -0.016) | 0.004 | Reference |
| WMH, cm^3^ |  |  |  |  |  |
| Model 1 | -0.024 (-0.448 to 0.401) | 0.912 | 0.093 (-0.130 to 0.317) | 0.412 | Reference |
| Model 2 | -0.096 (-0.519 to 0.327) | 0.657 | 0.154 (-0.103 to 0.412) | 0.239 | Reference |
| Model 3 | -0.039 (-0.497 to 0.418) | 0.865 | 0.160 (-0.104 to 0.425) | 0.234 | Reference |
| Abbreviations: *Aβ* beta-amyloid; *AD-CM* Alzheimer’s disease signature cerebral glucose metabolism; *OR* odds ratio; *CI* confidence interval.  Global Aβ retention, AV-1451, and WMH were used after natural log-transformation to achieve normal distribution.  Model 1 did not include any covariates, model 2 included age and sex as covariates, and model 3 included all potential covariates, including age, sex, education, apolipoprotein ε4 positivity, vascular risk score, body mass index, annual income status, occupational complexity, smoking, alcohol intake, vitamin B_12_, folate, platelet level, serum creatinine, and medication use. | | | | | |

| **SUPPLEMENTARY** **TABLE 5.** Results of multiple linear regression analyses including the interaction term between hemoglobin and age (or sex or education or apolipoprotein ε4 positivity or vascular risk score or clinical diagnosis or body mass index or annual income or occupational complexity) status predicting AD-CM for non-demented older subjects. | | |
| --- | --- | --- |
|  | B (95% CI) ^†^ | *P* |
| Hemoglobin | 0.024 (-0.067 to 0.116) | 0.600 |
| Age | <0.001 (-0.018 to 0.018) | 0.997 |
| Hemoglobin$\times$Age | -<0.001 (-0.001 to 0.001) | 0.890 |
|  |  |  |
| Hemoglobin | 0.021 (-0.015 to 0.056) | 0.262 |
| Sex | 0.008 (-0.307 to 0.322) | 0.962 |
| Hemoglobin$\times$Sex | -0.002 (-0.024 to 0.021) | 0.885 |
|  |  |  |
| Hemoglobin | 0.031 (0.006 to 0.056) | 0.015 |
| Education | 0.012 (-0.016 to 0.040) | 0.395 |
| Hemoglobin$\times$ Education | -0.001 (-0.003 to 0.001) | 0.235 |
|  |  |  |
| Hemoglobin | 0.030 (<0.001 to 0.061) | 0.053 |
| Apolipoprotein ε4 | 0.112 (-0.202 to 0.426) | 0.483 |
| Hemoglobin$\times$Apolipoprotein ε4 | -0.010 (-0.033 to 0.013) | 0.394 |
|  |  |  |
| Hemoglobin | 0.019 (0.003 to 0.035) | 0.020 |
| Vascular risk score | 0.003 (-0.125 to 0.131) | 0.960 |
| Hemoglobin$\times$Vascular risk score | -0.001 (-0.010 to 0.009) | 0.864 |
|  |  |  |
| Hemoglobin | 0.039 (0.008 to 0.070) | 0.014 |
| Clinical diagnosis | 0.151 (-0.134 to 0.437) | 0.299 |
| Hemoglobin$\times$ Clinical diagnosis | -0.015 (-0.036 to 0.006) | 0.151 |
|  |  |  |
| Hemoglobin | 0.072 (-0.006 to 0.149) | 0.071 |
| Body mass index | 0.027 (-0.017 to 0.070) | 0.228 |
| Hemoglobin$\times$ Body mass index | -0.002 (-0.005 to 0.001) | 0.170 |
|  |  |  |
| Hemoglobin | 0.014 (-0.008 to 0.036) | 0.207 |
| Annual income | -0.030 (-0.227 to 0.167) | 0.765 |
| Hemoglobin$\times$ Annual income | 0.003 (-0.011 to 0.017) | 0.659 |
|  |  |  |
| Hemoglobin | 0.016 (-0.004 to 0.035) | 0.115 |
| Occupational complexity | -0.013 (-0.108 to 0.082) | 0.791 |
| Hemoglobin$\times$ Occupational complexity | 0.001 (-0.006 to 0.008) | 0.772 |
| Abbreviations: *AD-CM* Alzheimer’s disease signature cerebral glucose metabolism; *CI* confidence interval.  ^†^Multiple linear regression model included hemoglobin, age (or sex or education or apolipoprotein ε4 positivity or vascular risk score or clinical diagnosis or body mass index or annual income or occupational complexity) and the interaction between hemoglobin and age (or sex or education or apolipoprotein ε4 positivity or vascular risk score or clinical diagnosis or body mass index or annual income or occupational complexity) treated as the independent variables; age, sex, education, apolipoprotein ε4, vascular risk score, clinical diagnosis, body mass index, annual income status, occupational complexity, smoking, alcohol intake, vitamin B_12_, folate, platelet level, serum creatinine, medication use, and declined food intake were treated as covariates; and AD-CM treated as the dependent variable. | | |
